# HOME ALONE REVISITED: FAMILY CAREGIVERS PROVIDING COMPLEX CARE

**DOI:** 10.1093/geroni/igz038.2740

**Published:** 2019-11-08

**Authors:** Susan Reinhard

**Affiliations:** AARP, Washington, District of Columbia, United States

## Abstract

In 2012, the Home Alone report brought to light the 46 percent of family caregivers that perform complex medical/nursing tasks. This presentation highlights new research conducted as part of a second Home Alone study to look closer at the family caregivers who perform these complex tasks with a specific focus on key medical/nursing tasks, including incontinence care, special diets, as well as certain sub-populations (e.g., multicultural communities, men, Millennials). An analysis of the increasing complexity of the challenges facing family caregivers who perform medical/nursing tasks in addition to assisting with instrumental activities of daily living and activities of daily living, will be shared. Presenters will also discuss its findings and the implications for individuals, their family caregivers and the healthcare providers who work with them.

